# Research Quality-Based Multivariate Modeling for Comparison of the Pharmacological Effects of Black and Red Ginseng

**DOI:** 10.3390/nu12092590

**Published:** 2020-08-26

**Authors:** Dong-Kyu Lee, Seongoh Park, Nguyen Phuoc Long, Jung Eun Min, Hyung Min Kim, Eugine Yang, Seul Ji Lee, Johan Lim, Sung Won Kwon

**Affiliations:** 1 College of Pharmacy, Seoul National University, Seoul 08826, Korea; dongqchicken@snu.ac.kr (D.-K.L.); phuoclong@snu.ac.kr (N.P.L.); mje0107@snu.ac.kr (J.E.M.); snuhmkim04@snu.ac.kr (H.M.K.); dltmfwl2@snu.ac.kr (S.J.L.); 2 Department of Statistics, Sungshin Women’s University, Seoul 02844, Korea; spark6@sungshin.ac.kr; 3 College of Pharmacy, Ewha Womans University, Seoul 03760, Korea; ginayang95@gmail.com; 4 Department of Statistics, Seoul National University, Seoul 08826, Korea; johanlim@snu.ac.kr; 5 Research Institute of Pharmaceutical Sciences, Seoul National University, Seoul 08826, Korea

**Keywords:** black ginseng, red ginseng, nonlinear principal component analysis, ginsenoside, pharmacological effect, systematic literature evaluation

## Abstract

Black ginseng has various pharmacological activities, but only few studies have compared its pharmacological effects with those of red ginseng. We conducted an integrative systematic literature evaluation and developed a non-inferiority test based on the multivariate modeling approach to compare the pharmacological effects of red ginseng and black ginseng. We searched reported studies on the pharmaceutical effects and composition of ginsenosides and assigned numeric scores using nonlinear principal component analysis, based on discretization measures for the included publications. Downstream weighted linear regression models were constructed to study the eight major biological activities that are generally known to be exhibited by red ginseng. Our statistical model, based on available ordinal information gathered from previous literature, helped in comparing the overlapping effects of black ginseng. Black ginseng showed antioxidant effects comparable to those of red ginseng; however, this variant was inferior to red ginseng in enhancing immunity, relieving fatigue, alleviating depression/anxiety, decreasing body fat, and reducing blood pressure. We have showed a cost-efficient method to indirectly evaluate the biological effects of ginseng products using data from published articles. This method can also be used to compare the nutritional and medicinal value of herbal medicines that share similar compositions of bioactive compounds.

## 1. Introduction

*Panax ginseng* species are one of the most important herbal medicines in Asian countries. Since the first modern scientific article of Petkov that reported its pharmacological effects in the 1950s, there have been more than 6000 reports describing the chemistry, biological effects, and medical use of *Panax ginseng* [1]. The health benefits and safety of this herbal medicine have been reported in vitro and in vivo, as well as in various clinical trials [2]. Intake of *Panax ginseng* and its products provides health benefits e.g., regulation of blood glucose [3] and blood pressure [4], improvement of the immune system [5], and fatigue relief [4,6]. The positive effects of *Panax ginseng* combined with anticancer drugs are widely reported in several in vitro and in vivo studies, indicating its role as a potential complementary treatment in patients with cancer [7]. Based on those unveiled pharmacological effects, more studies are in progress to better understand the impact of ginseng in medicine, including the specific roles of different ginseng variants. 

*Panax ginseng* or Korean ginseng are categorized into diverse variants including white, red, and black ginseng (Figure S1) and are present in various commercial products. The most popular variant, red ginseng, obtained by steaming, is generally regarded to have a broad and efficient spectrum of pharmacological effects compared with white ginseng [8]. These effects are due to the significant differences in not only the chemical constituents but also the ratios of the biologically active compounds. Red ginseng has been the most commonly used variant of ginseng for centuries, owing to its significant pharmacological effects that are evidence-based and supported by basic investigations and clinical studies [9]. Conversely, black ginseng is a relatively new product obtained by subjecting white ginseng to a special treatment that includes nine cycles of steaming [10]. The pharmacological effects of black ginseng have recently been acknowledged and more studies are rapidly emerging. Black ginseng extract, with respect to its ginsenoside composition, is found to show similarity in pharmacological effects with red ginseng [11,12,13,14]. For instance, studies have reported that black ginseng decreases cognitive deficits and exhibits anti-obesity and anti-inflammatory effects [13,15,16]. It also possesses potent antihyperglycemic effects and exerts its activity via modulating glucose metabolism in liver and muscle [17]. Moreover, black ginseng triggers the differentiation of myoblast and myotube hypertrophy. This observation suggests that black ginseng may help in preventing or treating muscle loss [18]. 

However, evaluation of the pharmacological effects of black ginseng in in vitro, in vivo, or clinical studies has been limited to a few models of diseases, and the results cannot be generalized for the practical use of this recent ginseng variant. Such generalization of the functions of herbal medicine has been mostly conducted by systematic review by rigorously collecting and assessing all available evidence [19,20]. This approach empirically evaluates whether pharmacological therapies can be facilitated for various types of diseases by summarizing the related studies. However, they are not equally reliable and sometimes can be biased because there is no feasible statistical method to indirectly evaluate pharmacological activity to date, while scoring the quality of the literature with consideration for the type of experiment, number of biomarkers, and other factors [21,22]. Especially, in the case of black ginseng, the limited evidence reduces the chances of being used as a herbal medicine, even though the potential function of such variant could be expected based on the shared bioactive components of the traditional variants and their effects. Therefore, a statistical model that can intermediate the compositional similarity of new variants to the function of traditional variants may facilitate the application of such new variants. 

This study proposes a quantitative research quality-based approach to appraise available evidence regarding the biological effects of black ginseng on eight major health ailments. Nonlinear principal component analysis (NLPCA) was used to assign a numeric score to every article based on discretized measures for journal articles. The optimized scoring system facilitated downstream analyses such as weighted linear regression, where scores were treated as weights of the study. This computational approach considered the study design, type of biological assays used in the study, and semiquantitative metrics of published articles (e.g., impact factor (IF) range) for modeling and comparison. Our novel method is a trust-based system to compare the nutritional and medical values of similar functional foods. Additionally, it is a cost-efficient method for future studies to further investigate ginseng products.

## 2. Materials and Methods 

**Figure 1:**
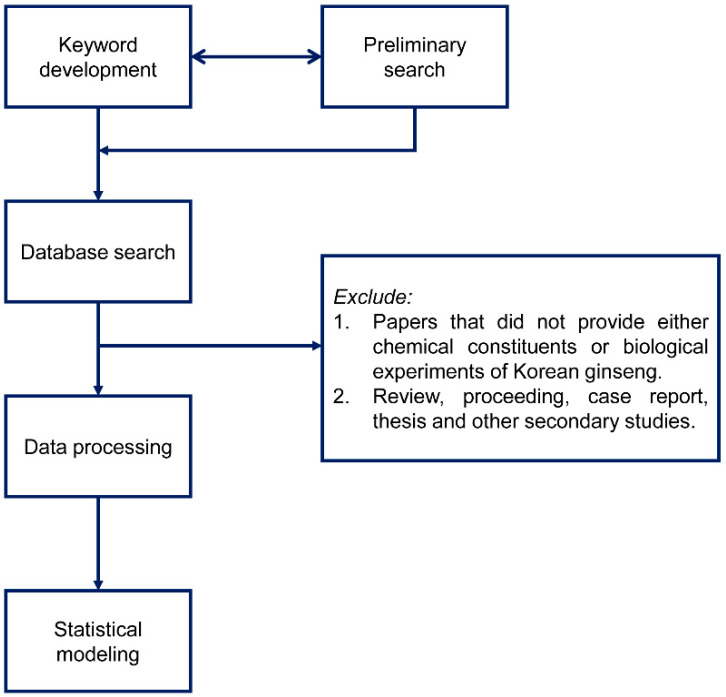
The workflow of the systematic literature evaluation and research quality-based evaluation.

The research workflow is depicted in Figure 1. After keyword development and preliminary search, we conducted an exhaustive literature search using PubMed, ScienceDirect, SCOPUS, and Google Scholar to gather all suitable articles to develop a statistical approach to compare the biological functions of red and black ginseng variants.

### 2.1. Literature Search

We conducted a literature search using a combination of keywords containing ginseng, ginsenoside, and eight medical benefits of ginseng [23,24,25]. In particular, for ginseng-related search terms, we used “white ginseng”, “red ginseng”, and “black ginseng”, but did not include *Insam* (Korean) and *ren shen* (Chinese). Twelve frequently reported ginsenosides including Rg1, Re, Rf, Rb1, Rc, Rb2, Rd, Rg2, Rh1, Rg3, Rk1, and Rg5 were used as search phrases. The ginsenoside list was decided based on a preliminary assessment of black and red ginseng-related studies. Regarding the medical benefits of ginseng, a combination of the following search terms was applied: “immunity” or “immunity boost” or “immune function”; “anti-fatigue” or “fatigue” or “anti-fatigue effect”; “anti-platelet” or “anti-platelet activity” or “platelet aggregation”; “memory” or “memory loss” or “cognitive improvement” or “cognitive;” “anti-oxidant” or “anti-oxidant activity”; “depression” or “anti-depressant effect”; “obesity” or “anti-obesity effect”; “blood pressure” or “hypertension”. Only original works of *Panax* species that selectively focused on *Panax ginseng* reported ginsenoside quantification and evaluated biological activity in vitro, in vivo, and in clinical trials were included in our study. We excluded review articles, proceedings, case reports, theses, and other secondary studies. We also excluded papers that did not provide chemical constituents or biological experiments pertaining to *Panax ginseng*. To assess the quality of articles and to remove duplicate articles, the authors read all the collected articles.

### 2.2. Data Extraction and Processing 

The following information was extracted from the included articles: active components (ginsenosides or whole extracts), publication year, journal, latest IF (version 2016 during the time of search), study types (in vitro, in vivo, human studies), and the tested assays. In addition, from the articles that reported the quantification of ginsenosides, we extracted the composition of major ginsenosides (mg/g) and calculated the mean and the standard deviation (SD). A list of recommended assays such as DPPH to measure antioxidant activity, etc., provided by the Ministry of Food and Drug Safety was used to evaluate applied bioassays. Next, the weighted score for each study was manually assigned and the hierarchy of evidence was set in the following order: in vitro < in vivo < human research. 

### 2.3. Statistical Modeling of Quality-Based Evaluation on Pharmacological Effects

Once the scoring system was established, we were able to gather n triplets $\left(b_{i},\;g_{i},\;s_{i}\right),\;i=1,\ldots ,\;n$. Here, $b_{i}$ is the number of bioassays used, $g_{i}$ is a ginseng type (0 = black, 1 = red), and $s_{i}\;is\;a\;$score of i-th research, respectively. It should be noted that when counting $b_{i}$, we fixed one of the eight functions of ginseng. Then, the weighted linear regression model was used to test our hypothesis comparing the number of bioassays used for red ginseng and black ginseng. To be specific, the model equation is represented by Equation (1):

(1) $$b_{i}=\mu +\Delta \;g_{i}+\;\epsilon_{i},\;\;\;\;\;\epsilon_{i}\sim N\left(0,\frac{\sigma^{2}}{s_{i}}\right),\;indep,\;i=1,\ldots ,\;n$$

where μ and μ+Δ are the average number of articles (or papers) from black and red ginseng, respectively, and $\sigma^{2}>0$ is the common variance across studies. By giving different weights to each research article, we assumed that even if two articles had the same number of assays, the results reported from a higher IF journal or from a human experiment were more reliable. Therefore, the quality of the paper and the experiment was considered, along with the number of bioassays conducted when comparing the two types of ginseng. Considering these notations, the claimed hypothesis was formally defined as per Equation (2), as follows:

(2) $$H_{0}:\;\mu =\gamma \left(\mu +\Delta \right)\;\;\mathrm{vs}.\;H_{1}:\;\mu >\gamma \left(\mu +\Delta \right)$$

with 0<γ<1 is a predetermined level of comparison. The value was set to 0.8, implying that to reject the null hypothesis, the mean (the number of papers) of black ginseng should be greater than 80% of that of red ginseng. The parameters μ, Δ, and $\sigma^{2}$ were easily estimated in the regression setting and the hypothesis testing was also followed by the classical theory in linear regression. After computing the *p*-value, we decided whether the data were strongly against the null hypothesis or not.

### 2.4. Reliability Scoring System for Ginsenoside Composition and Comparison of Activity 

We aimed to compare the average amount of ginsenosides or components (e.g., Rb1, Rg3, etc.,) in terms of a specific function between the two types of ginseng. We assumed to know the average amount of components, for example,$\;\{M_{1}^{b},\;M_{2}^{b},\ldots ,\;M_{G}^{b}\}$ for black ginseng and $\left\{M_{1}^{r},\;M_{2}^{r},\ldots ,\;M_{G}^{r}\right\}$ for red ginseng. We introduced a real-value quantitative measure that indicated an effect of a ginseng variant on its functionality. The statistic Q was defined specifically for ginseng variants denoted by type=b, r, and each category of health benefits denoted by fun (Equation (3)).

(3) $$Q\left(fun,\;type\right)=\overset{G}{\underset{g=1}{\sum }}w_{g}^{fun}M_{g}^{type}$$

where a weight $0\leq w_{g}^{fun}\leq 1$ reflected the relative impact size of ginsenoside g on health benefit fun. Thus, the functional activity of each ginseng was summarized using the weighted average Q(fun, b) for black ginseng and that of red ginseng by Q(fun r), which enabled a comparative analysis between the two variants. Since every component did not equally contribute to one specific health benefit, it was not appropriate to take an average of them with equal weights, i.e., $w_{g}^{fun}=\frac{1}{G},\;\forall g$. Thus, we again utilized the research-based scores.

The computational procedure for determining weights was based on the number of biomarkers used in research articles and was aggregated using the optimized scoring system. Construction of the scoring system used the same concept as described before; however, samples (or papers) were categorized according to the type of components or ginsenosides, type of functions, the IF of the journal, and type of experiment. After going through the optimization process for each functional benefit, we obtained quaternions $(b_{i},\;g_{i},\;s_{i}),\;i=1,\ldots ,\;n$, of the number of bioassays used, ginsenoside type, and the optimized score of i-th research for each given functionality. Subsequently, the weights for each functionality were determined using the following equation (Equation (4)):

(4) $$w_{g}^{fun}=\frac{\sum s_{i}b_{i}}{\sum_{i=1}^{n}s_{i}}$$

where the summation at the numerator runs over the set of articles where the type of ginsenoside and functionality are g and fun, respectively.

### 2.5. Description of the Optimization Problem in NLPCA Using Observations

To set up an optimization problem, we introduced the following variables: $f_{ij}$ was the number of articles in the i-th IF range, j-th was the experiment type; $s_{ij}≔s\left(i,j\right)$ was the score corresponding to the i-th IF range, j-th experiment type; and the total number of data n was equal to $\sum_{i,j}f_{ij}$. The score function of our interest was the solution of the following quadratic programming (Equation (5)):

(5) $$\underset{\left\{s_{ij}\right\}}{\max}\;\frac{1}{n}\underset{i,j}{\sum }f_{ij}s_{ij}^{2}-{\left(\frac{1}{n}\underset{i,j}{\sum }f_{ij}s_{ij}\right)}^{2}$$

subject to:

(1) $0\leq s_{ij}\leq 1$, for all i,j,
(2) $s_{t_{1}j}\leq s_{t_{2}j}$, $s_{it_{1}}\leq s_{it_{2}}$ if $t_{1}\leq t_{2}$ for all $i,j,t_{1},t_{2}$

Of note, the frequency $f_{ij}$ was given from data (and so was n) and there were I×J many variables $\left(s_{ij};1\leq i\leq I,\;1\leq j\leq J\right)$ that needed to be optimized. One can find that Equation (5) represents the maximization problem described in Section 3.3. Let $s_{t}=s\left(x_{t},\;y_{t}\right)$ be the score of the t-th sample having its IF $x_{t}$ and experiment type $y_{t}$. Then, the sample variance of ${\left\{s_{t}\right\}}_{t=1}^{n}$ is proportional to $\sum_{t=1}^{n}s_{t}^{2}/n-{\bar{s}}^{2}$, where $\bar{s}=\sum_{t=1}^{n}s_{t}/n$ is the sample mean of the scores. Considering $s_{t}$ is equal to one of the IJ scores, we have $\sum_{t=1}^{n}s_{t}=\sum_{i,j}f_{ij}s_{ij}$ and $\sum_{t=1}^{n}s_{t}^{2}=\sum_{i,j}f_{ij}s_{ij}^{2}$, which yields Equation (5).

### 2.6. Algorithm Implementation and Significance Level

All our algorithms were implemented using the R statistical software version 3.5.1 [26]. The significance level of a statistical test to reject the null hypothesis was set to 0.05.

## 3. Results

### 3.1. Descriptive Analysis of Included Studies

Based on our criteria, 477 ginseng-related articles were selected from more than 6000 records. The most commonly reported benefits of ginseng extracts and ginsenosides were immunity-enhancing and antioxidant effects, followed by effects related to memory enhancement, decrease in blood pressure, and decrease in body fat. Among the three types of ginseng products, black ginseng appeared to be the least reported product (Table 1). 

**Figure 2:**
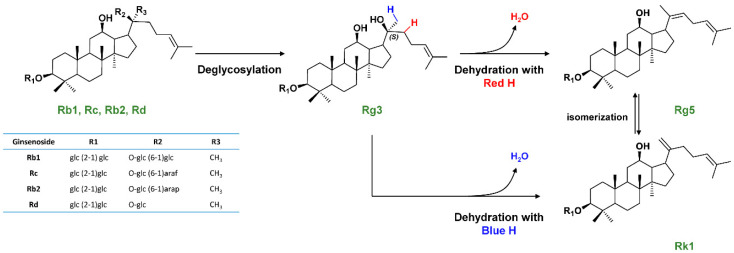
Ginsenoside conversion pathway among Rb1, Rc, Rb2, Rd, Rg3, Rg5, and Rk1.

Noticeably, there were no articles related to the effects of black ginseng extracts on alleviation of depression or anxiety. On the other hand, white ginseng and red ginseng were commonly reported to enhance immunity. Many articles reported that red ginseng particularly had blood-flow enhancing and antioxidant effects, while white ginseng was mostly reported to enhance memory and decrease blood pressure. The ginsenosides, specifically Rg1, Re, Rb1, Rd, and Rg3, received maximum interest. A representative conversion pathway of major ginsenosides during the heating process for producing red and black ginseng is given in Figure 2.

### 3.2. Ginsenoside Composition of Three Ginseng Variants and Selection of Comparison Target Variant against Black Ginseng Based on the Compositional Similarity

**Figure 3:**
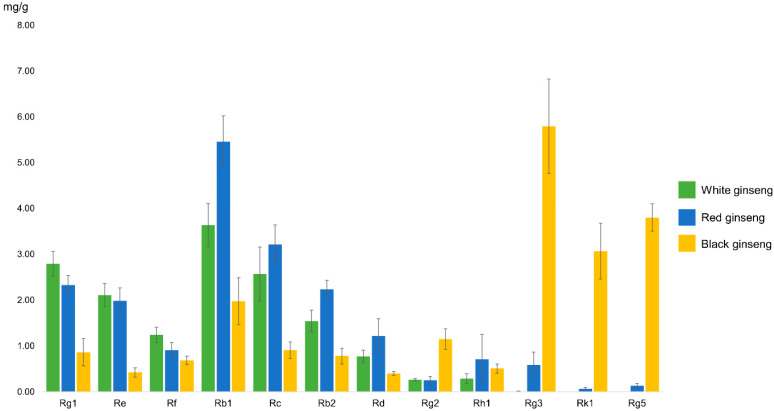
Twelve major ginsenoside constituents of white ginseng, red ginseng, and black ginseng.

Among the included articles, there were 10 articles that reported the ginsenoside composition of three ginseng variants [10,12,27,28,29,30,31,32,33,34]. Among ginsenosides, Rg3 (5.79 ± 3.27 mg/g, n = 6), Rg5 (3.80 ± 0.67 mg/g, n = 3), and Rk1 (3.07 ± 1.37 mg/g, n = 4) are the major components of black ginseng (Figure 3). The major components of red ginseng are Rb1 (5.46 ± 2.54 mg/g, n = 6), Rc (3.21 ± 1.89 mg/g, n = 6), Rg1 (2.33 ± 0.87 mg/g, n = 6), and Rb2 (2.23 ± 0.87 mg/g, n = 6). Similarly, white ginseng contains mostly Rb1 (3.64 ± 1.55 mg/g, n = 8), Rg1 (2.79 ± 0.87 mg/g, n = 8), Rc (2.57 ± 1.96 mg/g, n = 8), and Rb2 (1.54 ± 0.79 mg/g, n = 8), although the absolute values are different from those of red ginseng.

The ginsenoside compositional similarity of black ginseng to two traditional (white and red) ginseng variants was investigated to generate a functionality scoring system for evaluating the comparable pharmacological effects. Heat processing of raw ginseng into red and black ginseng was proved to produce ginsenosides Rg3, Rk1, and Rg5 (Figure 3) by deglycosylation and subsequent dehydration, whereas white ginseng contained no such ginsenosides. It is worth mentioning that Rg3, Rk1, and Rg5 are important components responsible for the pharmacological effect of ginseng variants, as these heat-induced ginsenosides were reported in 33 papers on the function of each single isolated component. Therefore, multivariate modeling was used to investigate whether black ginseng possesses the overlapping effect of the eight major biological activities of red ginseng. 

### 3.3. Functionality Scoring System for Comparison of Ginseng Type 

In this study, the quality of an article was determined based on the IF of the journal (when the article was published), the quality of the experiment according to the experiment type (in vitro, in vivo, or clinical (human) research), and the number of bioassays used. For example, Wang et al. [35] reported that the antioxidant effect of Rg3 could play a protective role against adriamycin-induced cardiotoxicity. Even though they performed both in vivo and in vitro studies, only the in vitro study included commonly used antioxidant assays. Statistically significant results of superoxide dismutase, reactive oxygen species, malondialdehyde, and glutathione peroxidase-1 were shown to be associated with alleviation of adriamycin-induced cytotoxicity in endothelial cells. Thus, this one study suggested four biomarkers for use in an in vitro assay, and this value facilitated into the scoring system together with the IF of the Phytomedicine journal. 

However, the type of experiment did not give a measurable score for each level, though it is generally accepted that clinical research is more reliable than in vivo studies, which, in turn, are considered more reliable than in vitro studies. Furthermore, the number represented by IF may not quantify the subjective importance of the article according to the researchers. For example, a difference of 5 points in the IF of two journals whose scores range around 5 should be treated differently than the same difference in case of journals with IFs around 20. Therefore, an optimal nonlinear transformation was required to map the multidimensional data (ordinal variables in our case) into a numeric value (a continuous score in (0,1) in our case). This transformation process can be performed using optimal scaling methods, which have often been explored in statistics [36,37]. Among the existing methods, we used NLPCA to establish a new scoring system under the constraints that ordinal variables should satisfy [38]. However, the usual NLPCA aims to find a scoring mechanism for each ordinal variable independently, which is not the case for this paper, as we needed a mechanism that preserves not only marginal ordering but also joint ordering. This motivated us to develop our own scoring system. To discuss our method more rigorously, X was denoted as an ordinal variable corresponding to the discretized IF (with prespecified bins) and Y the type of experiment. Assuming that X had levels in {1, …, I} and Y in {1, …, J} with increasing order, the scoring mechanism S, of two ordinal variables (X,Y) should satisfy the following:

(1) 0≤S(x,y)≤1, x∈{1, …, I}, y∈{1, …, J},
(2) S is order-preserving; $S\left(t_{1},y\right)\leq S\left(t_{2},y\right)$ and $S\left(x,\;t_{1}\right)\leq S\left(x,\;t_{2}\right)$ if $t_{1}\leq t_{2}$ for any x,y.

These conditions were obvious because the score was defined over (0, 1) and should reflect the ordering of ordinal levels. The usual NLPCA aims to find a scoring mechanism for each ordinal variable, but we additionally consider a quantification that preserves joint ordering. We aimed to find, among the possible systems with the two conditions, the optimal scoring method S that can best explain its dispersion:

$$\underset{s}{\max}Var\left(S\left(X,Y\right)\right).$$

This optimization problem was not new because if S were a linear function, then this problem returned to the usual PCA. The sample version of it is represented by the quadratic programming (QP), which can be easily solved via standard optimization tools (e.g., the Nelder–Mead method).

### 3.4. Comparison of the Pharmacological Activities of Black and Red Ginseng Extracts Using the Weighted Linear Regression with the Scoring System Optimized by NLPCA

In total, 168 articles related to red ginseng and black ginseng extracts were categorized based on the experiment type, the number of bioassays, and IF (if available) of the journals that these studies were published in. Before applying the optimal scaling method to obtain the optimal score system, we binned continuous IFs into five groups, as shown in Table S1. We used NLPCA and obtained optimized scores that satisfied the two targeted properties. Table S1 shows that the reliability of a study heavily depended on the type of experiments performed, and consequently, the score of in vivo (or in vitro) research in the highest IF group was lower than that of human (or in vivo) research in the lowest IF group.

Using a significance level of 0.05 in all functions except that for memory, there was not enough evidence to reject the null hypothesis (Equation (2)) to indicate that the number of indicators for black ginseng extract was comparable to at least 80% of that of red ginseng (Table 2). Hence, it was difficult to state whether black ginseng had the same functions as that of red ginseng. As no research has been conducted on black ginseng in terms of its antidepressant/antianxiety activity, the linear regression model in (1) could not be estimated, and thus, hypothesis testing was not possible for this pharmacological effect. Conversely, in terms of memory enhancement, the number of bioassays from black ginseng research was comparable to 80% of that of red ginseng. Therefore, we were able to verify the potential for memory enhancement function in black ginseng extract.

### 3.5. Evaluation of the Pharmacological Activities Dependent on Individual Ginsenosides using Metrics-Based Research Quality

In the second analysis, we initially investigated the average content of ginsenosides in black ginseng and red ginseng using articles that reported the concentrations of these components in ginseng. Since it is known that individual ginsenosides have different health benefits, we calculated their relevant weights to compute overall activity enhanced by ginseng intake. The optimized scoring system for articles is tabulated in Table S2.

Next, the remaining computation was straightforward and involved obtaining a pair of estimates of (Q(fun, b), Q(fun, r)) for every health benefit, as shown in the last row of Table 3. Contrary to the analysis of individual extracts, the scoring system with the composition of individual ginsenosides found that black ginseng was highly potent in enhancing immunity. While the improvement in immunity function was primarily due to Rb1 in red ginseng, Rg3 was responsible for this effect in black ginseng. In the case of fatigue relief, we obtained similar results for black and red ginseng, even though fewer papers reported such an effect. Further, we verified that Rg3 and Rb1 in black and red ginseng were highly responsible for lowering blood pressure. Studies report only a few ginsenosides were involved in controlling blood pressure; hence, these were not included in the evaluation of the functions of red ginseng. We expected that the effect would be less pronounced for black ginseng. Rb1 in red ginseng also showed a much greater effect in lowering body fat. To date, the lowering of body fat is not reported with the use of red ginseng. In the case of the antioxidant effect, the reliability in the functions of black and red ginseng was almost the same. While Rg3 was the major component in black ginseng responsible for the antioxidant effect, several compounds collectively contributed to this effect in red ginseng. Black ginseng exhibited minor antidepressant activity, while this was not observed in the case of red ginseng. Black ginseng showed a more reliable memory-boosting ability than red ginseng. This effect was shown to be mainly caused by Rg5, the relatively abundant component in black ginseng. It is highly probable that black ginseng markedly improves cognitive ability. Black ginseng was more efficacious than red ginseng in improving blood flow. The compound Rg3 appears to be involved in bringing about this effect. In addition, Rk1 was also found to be an important component for such a pharmacological effect, which is the major component of black ginseng compared with other variants. 

## 4. Discussion

In the current study, we developed a method to indirectly determine the biological effects of black ginseng by systematically comparing this relatively new variant with red ginseng, the commonly used *Panax ginseng* variant. Based on the studies on ginseng extracts, the biological effects of black ginseng cannot be proven to be comparable to those of red ginseng except for the memory enhancement effect (Table 2). NLPCA results with integrative scoring of the quality and number of papers on red ginseng supported that the traditional variant was more credible than black ginseng. Because the optimal scoring system based on the literature on the effect of ginseng extract did not consider the composition of ginsenosides, the major active components of ginseng, it is natural for the traditional variants to have a higher score, as they have been used and studied for longer periods. 

When it comes to pharmacological activity evaluation based on individual ginsenosides using metrics-based research quality, black ginseng showed potential pharmacological effects in a total of eight categories. The optimized scoring system suggested that the immunomodulation, fatigue and blood pressure regulation, cognitive improvement, and antioxidant activities of black ginseng were statistically comparable to those of red ginseng. Based on comparative analysis, our quantitative analysis results suggested that black ginseng has similar efficacy to red ginseng in enhancing immune responses, owing to its significantly higher amount of Rg3. In addition, black ginseng showed a higher impact than red ginseng in enhancing memory. This effect may be attributed to the significantly higher level of Rg5 and Rg3 in black ginseng [39]. Moreover, Rg3 in black ginseng was more effective in improving blood flow than that in red ginseng. Furthermore, the advantages of black ginseng were reflected via its significant effects in enhancing blood flow and memory. Lastly, black ginseng showed antioxidant effects comparable to those of red ginseng, while red ginseng was more efficacious in enhancing immunity, relieving fatigue, depression, and anxiety, and reducing body fat and blood pressure. Collectively, individual ginsenoside-based comparative studies supported that the higher amounts of Rg3, Rk1, and Rg5 in black ginseng contribute to its biological activities as a nutraceutical. However, there is a limitation that our optimal scoring system cannot directly determine whether black ginseng has better efficacy than the traditional variant. For this, further studies should be conducted. Nevertheless, our approach revealed that black ginseng, a new variant, could be used for some pharmacological effects and could be worth investigating in future studies. 

Our novel statistical model enhances the process of using available ordinal information gathered from previous literature and helps in measuring and comparing the impacts of similar types of herbal medicines. The core database can be easily updated upon the availability of new research articles, and the new scoring system can be applied with relative ease. Specifically, this system is highly practical for comparing the pharmacological effects of a newly developed variant or species of some medicinal plants. If a change in some factors, including cultivation or post-processing of medicinal plants, is found to lead to compositional differences in bioactive compounds, it is possible to estimate whether the effect of the modified variant is comparable to that of the original. Because this statistical model does not have over or underrepresentation of a few limited findings, publication bias, which is generally considered as the limitation of conventional systematic review or meta-analysis, could be prevented [40,41,42]. However, all the above results are based on the calculated estimates of the composition of bioactive components and the function of each. Hence, further research delineating the functions of a new variant is required to validate these findings. An additional problem is the limited applicability of this statistical model for comparing the variants which have similar bioactive components. Even though red and black ginseng are produced from the treatment of white ginseng, huge alteration of the ginsenoside occurred in those two variants, and thus, restricted the comparison of those with white ginseng. Further refining of the statistical model would extend its applicability to the broad subtypes of herbal medicine with higher compositional differences.

One limitation of our study is that statistical estimation may not be accurate when adequate evidence is not available. For example, substantial evidence regarding the anti-obesity effects of ginseng has been obtained from in vivo experiments; hence, an extrapolation of these results in humans would not be possible [25]. The biological justification for the formula “a sum of the weighted number of biomarkers × amount of ginsenoside”, representing the functional activity, is also a matter of concern. The number of biomarkers could make the result much more credible, but it cannot be easily converted to the numerical estimates. Likewise, the amount of bioactive component, ginsenoside in this paper, could not be linearly correlated in some cases. Even though our systematic literature evaluation with multivariate modeling could collectively integrate the information about herbal medicines from the literature, substantial testing for comparing the pharmacological activity would be required. To objectively compare the functions of black ginseng and red ginseng, it is essential to produce the two types of ginseng from the same raw material under identical conditions. Finally, this modeling not only excludes the possibility of the integrative effect of ginsenosides but also ignores the effect of minor ginsenosides or other active ingredients such as polysaccharides [43].

## 5. Conclusions

We conducted a systematic literature evaluation and extracted quantitative data for multivariate modeling to compare the pharmacological effects of black and red ginseng, using a quality-based evaluation of research articles. The scoring system with statistical significance improved the estimation of the functions, while reducing the limitation of the conventional systematic review. Our results suggest that black ginseng, a recent trending product with few publications regarding its activities, may have comparable biological activities to red ginseng with respect to improving immunity, memory, and fatigue, and exhibiting antioxidant effects. We believe this systematic evaluation approach could identify new perspectives of black ginseng or some minor variant of ginseng in the future. This could further our understanding of the herbal medicines that are not generally used in modern medicine.

## Figures and Tables

**Table 1:** The number of collected papers considering three types of ginseng extracts and eight individual ginsenosides.

Function	White Ginseng	Red Ginseng	Black Ginseng	Rg1	Re	Rf	Rb1	Rc	Rb2	Rd	Rg2	Rh1	Rg3	Rk1	Rg5	Count
Enhanced Immunity	48	37	3	18	11	1	15	2	2	10	0	5	5	2	4	163
Fatigue Relief	6	9	8	0	0	0	3	0	0	1	0	0	5	0	0	32
Enhanced Blood Flow	2	20	1	4	0	0	0	0	0	1	2	0	3	0	0	33
Enhanced Memory	16	4	7	9	1	0	9	0	0	2	1	2	1	0	1	53
Antioxidant	9	29	8	6	5	0	1	0	1	6	1	1	12	2	3	84
Antidepressant/Antianxiety	9	2	0	4	1	0	3	0	0	0	0	0	0	0	0	19
Decreased Body Fat	5	15	3	4	3	0	5	2	2	1	0	1	2	0	0	43
Decreased Blood Pressure	18	16	1	3	0	0	4	0	0	2	0	1	5	0	0	50
															Total	477

**Table 2:** *p*-value summarization among eight compared pharmacological effects between black ginseng extract and red ginseng extract.

Function	*p*-Value
Enhanced immunity	0.386
Fatigue Relief	0.661
Enhanced Blood Flow	0.419
Enhanced Memory	0.021
Antioxidant	0.755
Antidepressant/Antianxiety	-
Decreased Body Fat	0.159
Decreased Blood Pressure	0.205

**Table 3:** Weighted average amount of individual ginsenosides between black ginseng and red ginseng among eight compared pharmacological effects.

Compound	Enhanced Immunity		Fatigue Relief		Enhanced Blood Flow		Enhanced Memory		Antioxidant		Antidepressant/Antianxiety		Decreased Body Fat		Decreased Blood Pressure	
	BG	RG	BG	RG	BG	RG	BG	RG	BG	RG	BG	RG	BG	RG	BG	RG
Rb1	7.69	20.36	6.30	16.68	-	-	2.73	7.22	1.97	5.22	3.23	8.55	10.00	26.46	2.55	6.75
Rb2	1.06	3.19	-	-	-	-	-	-	0.78	2.34	-	-	1.21	3.65	-	-
Rc	2.69	8.89	-	-	-	-	-	-	-	-	-	-	1.30	4.31	-	-
Rd	1.57	5.76	1.59	5.82	1.19	4.36	0.39	1.45	2.20	8.07	-	-	0.79	2.91	0.39	1.45
Re	0.95	8.20	-	-	-	-	0.25	2.15	0.32	2.82	1.00	8.62	0.46	3.96	-	-
Rf	2.38	4.61	-	-	-	-	-	-	-	-	-	-	-	-	-	-
Rg1	2.19	9.34	-	-	0.70	3.00	0.80	3.42	1.70	7.26	1.07	4.59	1.79	7.64	2.02	8.62
Rg2	-	-	-	-	1.77	0.54	0.68	0.21	2.75	0.85	-	-	-	-	-	-
Rg3	24.73	3.39	13.82	1.90	11.13	1.53	9.74	1.33	13.90	1.91	-	-	9.43	1.29	7.97	1.09
Rg5	5.69	0.32	-	-	-	-	11.40	0.64	2.33	0.13	-	-	-	-	-	-
Rh1	1.56	2.45	-	-	-	-	0.72	1.12	1.41	2.21	-	-	0.70	1.10	0.70	1.10
Rk1	2.12	0.06	-	-	3.06	0.08	-	-	2.12	0.06	-	-	-	-	-	-
Total	52.68	66.61	21.72	24.40	17.87	9.54	26.73	17.58	29.53	30.89	5.32	21.77	25.72	51.36	13.65	19.03

Abbreviations: BG—black ginseng; RG—red ginseng.

## Supplementary Materials

The following are available online at https://www.mdpi.com/2072-6643/12/9/2590/s1, Figure S1: Photographs of white (a), red (b) and black (c) ginseng, Table S1: Optimized scoring system obtained from nonlinear principal component analysis of articles related to black and red ginseng extract, Table S2: Optimized scoring system obtained from nonlinear principal component analysis of articles related to individual ginsenosides.
