# Supplementary material for: Research Quality-Based Multivariate Modeling for Comparison of the Pharmacological Effects of Black and Red Ginseng
Source: Nutrients. 2020 Aug 26;12(9):2590. doi: 10.3390/nu12092590 (PMC7551003; doi:10.3390/nu12092590)
Supplement: Supplementary file 1 [file nutrients-12-02590-s001.pdf]

Research Quality-Based Multivariate Modeling for Comparison of the Pharmacological Effects of Black and Red Ginseng

Dong-Kyu Lee [1,†], Seongoh Park [2,†], Nguyen Phuoc Long [1], Jung Eun Min [1], Hyung Min Kim [1], Eugine Yang [3], Seul Ji Lee [1], Johan Lim [4] and Sung Won Kwon [1,5,*]

[1] College of Pharmacy, Seoul National University, Seoul 08826, Korea; dongqchicken@snu.ac.kr (D.-K.L.); phuoclong@snu.ac.kr (N.P.L.); mje0107@snu.ac.kr (J.E.M.); snuhmkim04@snu.ac.kr (H.M.K.); dltmfwl2@snu.ac.kr (S.J.L.)

[2] Department of Statistics, Sungshin Women's University, Seoul 02844, Korea; spark6@sungshin.ac.kr (S.P.)

[3] College of Pharmacy, Ewha Womans University, Seoul 03760, Korea; ginayang95@gmail.com (E.Y)

[4] Department of Statistics, Seoul National University, Seoul 08826, Korea; johanlim@snu.ac.kr (J.L.)

* Correspondence: swkwon@snu.ac.kr; Tel.: +82-2-880-7844; Fax: +82-886-7844

† These authors contributed equally to this work.

Figure S1. Photographs of white (a), red (b) and black (c) ginseng

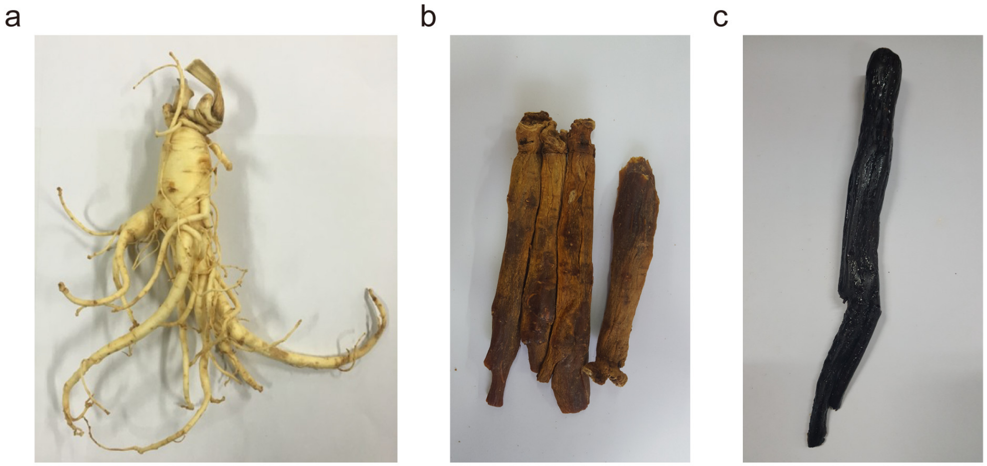

Table S1. Optimized scoring system obtained from nonlinear principal component analysis of articles related to black and red ginseng extract

Categorized IF		(0, 1.29)	(1.29, 2.69)	(2.69, 3.42)	(3.42, 3.9)	(3.9, 5.99)
	**Type of experiment**					
	In vitro	0.101	0.101	0.104	0.113	0.128
	In vivo	0.203	0.205	0.228	0.299	0.4
	Human	1	1	1	1	1

Abbreviation: IF, impact factor.

Table S2. Optimized scoring system obtained from nonlinear principal component analysis of articles related to individual ginsenosides

**Categorized IF**	**Type of experiment**	(0, 1.56)	(1.56, 1.76)	(1.76, 2.22)	(2.22, 2.54)	(2.54, 2.56)	(2.56, 2.73)	(2.73, 3.23)	(3.23, 3.9)	(3.9, 8.78)
	In vitro	0.101	0.105	0.128	0.134	0.151	0.160	0.171	0.181	0.192
	In vivo	0.220	0.241	0.263	0.284	0.304	0.324	0.345	0.365	0.385
	Human	0.559	0.608	0.659	0.711	0.761	0.811	0.862	0.913	0.963

Abbreviation: IF, impact factor.
